# A fat body-derived apical extracellular matrix enzyme is transported to the tracheal lumen and is required for tube morphogenesis in *Drosophila*

**DOI:** 10.1242/dev.109975

**Published:** 2014-11

**Authors:** Bo Dong, Guangxia Miao, Shigeo Hayashi

**Affiliations:** 1 Laboratory for Morphogenetic Signaling, RIKEN Center for Developmental Biology, 2-2-3 Minatojima-minamimachi, Chuo-ku, Kobe, Hyogo 650-0047, Japan; 2 Department of Biology, Kobe University Graduate School of Science, 1-1 Rokkodai-cho, Nada-ku, Kobe, Hyogo 657-8051, Japan

**Keywords:** Fat body, Chitin, Transcytosis, *Drosophila* trachea

## Abstract

The apical extracellular matrix plays a central role in epithelial tube morphogenesis. In the *Drosophila* tracheal system, Serpentine (Serp), a secreted chitin deacetylase expressed by the tracheal cells plays a key role in regulating tube length. Here, we show that the fly fat body, which is functionally equivalent to the mammalian liver, also contributes to tracheal morphogenesis. Serp was expressed by the fat body, and the secreted Serp was taken up by the tracheal cells and translocated to the lumen to functionally support normal tracheal development. This process was defective in *rab9* and *shrub/vps32* mutants and in wild-type embryos treated with a secretory pathway inhibitor, leading to an abundant accumulation of Serp in the fat body. We demonstrated that fat body-derived Serp reached the tracheal lumen after establishment of epithelial barrier function and was retained in the lumen in a chitin synthase-dependent manner. Our results thus reveal that the fat body, a mesodermal organ, actively contributes to tracheal development.

## INTRODUCTION

Organ systems are functionally coupled by the exchange of essential humoral factors, including hormones, nutrients and metabolites that coordinate organismal homeostasis. In vertebrates, the liver plays a central role in providing nutrients and proteins required by other organs, as well as detoxifying unfavourable metabolites (Arias, 2009). The fat body of insects is functionally equivalent to the mammalian liver and is the major storage site of lipids, proteins and sugars, thereby serving as an energy reservoir (Chapman, 1998).

In order for proteins synthesized in one organ to function in another, they must be released to the haemolymph or bloodstream, taken up by the target tissues and delivered to the correct cellular compartments. Transcytosis is a key process that facilitates protein translocation across epithelial barriers. In mammals, the polymeric immunoglobulin IgA receptor is transcytosed from the basal of the intestinal epithelium to the gut lumen (Apodaca et al., 1994; Rojas and Apodaca, 2002).

Insect extracellular matrix (ECM) consists of the polysaccharide chitin and an assortment of proteins required for exoskeleton assembly and tracheal development. For example, serpentine (Serp) is a chitin deacetylase required for the tracheal tube morphogenesis (Luschnig et al., 2006). Both the overexpression and loss of *serp* function result in trachea with excessively long and convoluted tubes (Luschnig et al., 2006; Wang et al., 2006). However, whether non-epithelial tissues contribute to tracheal tubulogenesis is unknown.

Here, we show evidence suggesting that the fat body serves as a source of Serp involved in tracheal development. Serp was expressed in the fat body and the secreted Serp in the haemolymph was transported across epithelial barriers into the tracheal lumen. Knock-down of non-tracheal *serp* expression resulted in an abnormal tracheal tube morphology during embryonic and larval stages. Thus, long-range transport of Serp through the haemolymph permits systemic control of respiratory tube morphogenesis.

## RESULTS AND DISCUSSION

### Serp synthesis and trafficking through the fat body

**Fig. 1.**
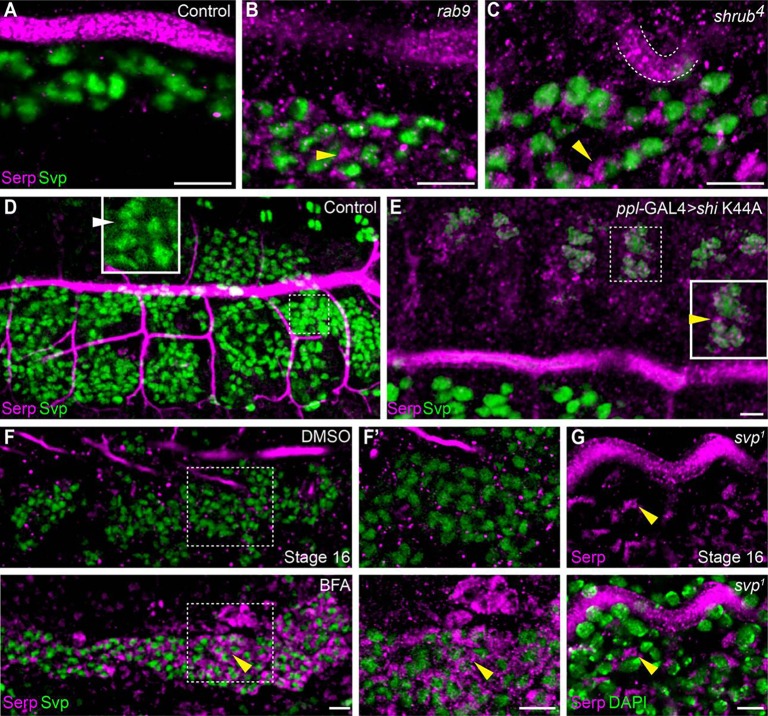
**Serpentine accumulation in the fat body cells of vesicle transport mutants and drug-treated embryos.** (A-C) Stage 16 control (A), *rab9* (B) and *shrub* (C) homozygous mutant embryos stained with anti-Serp and anti-Seven-up (Svp). Yellow arrowheads in B and C indicate strong Serp accumulation in the fat body cells. (D,E) Stage 16 control and *ppl-*GAL4-*shi* K44A-expressing embryos stained with anti-Serp and anti-Svp. Yellow arrowhead in E indicates Serp accumulation in Svp-positive cells. (F,F′) Embryos treated with DMSO (top panel) and brefeldin A (BFA) (bottom panel). BFA caused substantial amounts of Serp to be retained within the fat body cells (yellow arrowheads in F and F′). (G) Stage 16 *svp^1^* mutant embryos stained with anti-Serp antibody (top) and counter staining with DAPI (bottom). Yellow arrowhead indicates Serp accumulation in mesodermal cells. Scale bars: 10 μm.

The small GTPase Rab9 mediates Serp sorting and retrograde trafficking, and regulates tracheal tube geometry (Dong et al., 2013). The intracellular trafficking and luminal deposition of Serp is also influenced by Vps32/Shrub, a component of ESCRT III (endosomal sorting complex required for transport III) (Dong et al., 2014). We found abundant Serp protein in the fat bodies of *rab9* and *shrub* mutants (Fig. 1A-C), while its level in the tracheal lumen of these mutants was reduced. Other luminal proteins did not show fat body accumulation in the mutant embryos (supplementary material Fig. S1A,B). To determine whether *shrub* functions cell-autonomously in the fat body, we expressed a dominant-negative Shrub GFP (Sweeney et al., 2006) using fat body-specific drivers (see Materials and Methods). Regions of partially collapsed tracheal tube were observed in 31% (*n*=50) of third-instar larvae (supplementary material Fig. S2), suggesting that fat body contributes to normal tracheal development.

The fat body-accumulated Serp in *rab9* and *shrub* mutants could be derived from fat body cells or transported from another source through endocytosis. To examine these possibilities, we blocked clathrin-dependent endocytosis in the fat body using a dominant-negative form of Dynamin, *shi* K44A, using the *ppl-*GAL4 fat body driver. In control embryos, Serp was normally deposited in the tracheal lumen and it was not detected in fat body cells (Fig. 1D). However, in *shi* K44A-expressing embryos, Serp accumulated in intracellular puncta in the cells positive for the fat body marker Seven-up (Svp) (Fig. 1E). The level of Serp accumulation in the fat body cells was lower than in *rab9* and *shrub* mutants (compare inset in Fig. 1E with Fig. 1B,C). Dynamin inhibition is known to interfere indirectly with exocytosis (Koenig et al., 1983). Thus, this result suggests that Serp accumulates in fat body cells with reduced endocytic and exocytic activity. To reduce exocytic activity, we abrogated protein trafficking from the endoplasmic reticulum to the Golgi using brefeldin A (BFA) (Misumi et al., 1986). BFA treatment resulted in strong Serp accumulation in the fat body (Fig. 1F,F′). These results indicated that Serp protein accumulation in the fat bodies of *rab9* and *shrub* mutants was due to cell-intrinsic Serp expression.

The *serp* transcript is present in the pupal fat body (FlyBase), and we confirmed that the *serp* transcript was expressed in the tracheal system from stages 12 to 15, followed by strong expression in the epidermis by stage 16 (Luschnig et al., 2006) (supplementary material Fig. S3A). Low-level *serp* expression was also detected in the stage 16 fat body (yellow arrowheads in supplementary material Fig. S3B).

We also examined the fat body in mutants of the transcription factor Svp, which is required for fat body cell differentiation (Hoshizaki et al., 1994). Fat body cells in *svp1* mutants accumulated Serp (Fig. 1G), suggesting that Svp function is required for fat body release of Serp. Collectively, these findings suggest that Serp protein is expressed in the fat body.

### Serp translocates from the haemolymph to the tracheal lumen

**Fig. 2.**
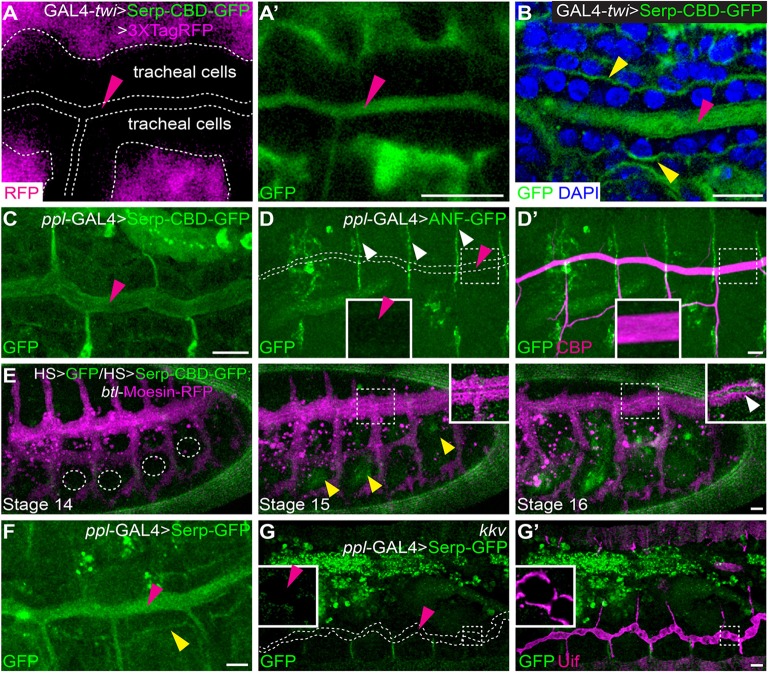
**Translocation of Serp from haemolymph to the tracheal lumen.** (A,A′) Confocal sections of living stage 14 embryos expressing Serp-CBD-GFP and TagRFP by *twi-*GAL4. Only GFP was detected in the tracheal lumen. (B) Confocal sections of fixed stage 16 embryos expressing Serp-CBD-GFP stained with DAPI. Magenta and yellow arrowheads in A-B indicate Serp-CBD-GFP deposition in the tracheal lumen and the basal surface of tracheal cells, respectively. (C) Confocal section of fixed stage 16 embryos expressing Serp-CBD-GFP by *ppl*-GAL4. The magenta arrowhead indicates GFP signal present in the tracheal lumen. (D,D′) Fixed embryos expressing ANF-GFP by *ppl*-GAL4 were stained with CBP. Magenta arrowheads indicate the absence of ANF-GFP from the tracheal lumen. White arrowheads indicate the segmental boundaries. (E) Induction of Serp-CBD-GFP expression at stage 15 by localized laser heat shock. Left, middle and right panels show three stages of a time lapse series. White dashed circles indicate the heat-shock positions. Yellow arrowheads indicate GFP signal in the heat-shocked position at stage 15 (expressed from HS>GFP). White arrowhead in inset indicates GFP signal present in the tracheal lumen at stage 16. (F) Confocal sections of living embryos expressing full-length Serp-GFP by *ppl*-GAL4. The magenta arrowhead indicates Serp-GFP in the tracheal lumen. Yellow arrowhead indicates Serp-GFP accumulation at the basal surface of tracheal cells. (G,G′) *ppl*-GAL4>Serp-GFP expressing stage-16 *kkv* mutant embryos were stained with anti-Uif. Magenta arrowheads indicate the absence of GFP signal in the tracheal tube. Scale bars: 10 μm.

Serp does not normally accumulate in the fat body. Thus, we postulated that fat body-derived Serp is secreted into the haemolymph, taken up by tracheal cells and delivered to the tracheal lumen. To test this hypothesis, we used a fusion protein containing the chitin-binding domain of Serp (Serp-CBD-GFP), which is a secreted luminal-marker (Luschnig et al., 2006). Serp-CBD-GFP was expressed using *twi-*GAL4. The co-expressed cytoplasmic TagRFP marker showed that the *twi-*GAL4 driver was active in the mesodermal, but not tracheal, cells (Fig. 2A). By contrast, Serp-CBD-GFP was present in the mesoderm and in the tracheal lumen (Fig. 2A,A′). Serp-CBD-GFP also accumulated at the basal surface (Fig. 2B). We next expressed Serp-CBD-GFP using driver *ppl*-GAL4 and found its accumulation in the tracheal lumen (Fig. 2C). By contrast, a functionally inert secreted protein marker, ANF-GFP (Rao et al., 2001; Tsarouhas et al., 2007), with a similar molecular weight and diffusion characteristics as Serp-CBD-GFP (Dong et al., 2014) was absent from the tracheal lumen (magenta arrowhead in Fig. 2D,D′).

These experiments suggest that Serp translocates to the tracheal lumen. Transcytosis could be the mechanism for Serp translocation; however, it is also possible that fat body-derived Serp reaches the tracheal tube through protein diffusion before trachea acquires epithelial barrier function in the middle of tracheal tubulogenesis (stage 15) (Paul et al., 2003). To distinguish these two possibilities, we induced expression of Serp-CBD-GFP in mesodermal cells surrounding the trachea at stage 15 using infrared laser-induced local heat shock (Kamei et al., 2009; Miao and Hayashi, 2014), and found GFP signal in the tracheal lumen at stage 16 (Fig. 2E, supplementary material Movie 1). This indicates that Serp-CBD-GFP can reach the tracheal lumen across epithelial barriers through transcytosis.

Next, we asked about the role of chitin in Serp transport. Full-length Serp fusion protein (Serp-GFP) expressed by *ppl-*GAL4 accumulated in the tracheal lumen and the basement membrane of tracheal cells (Fig. 2F). When the experiment was repeated in mutants of chitin synthase, *krotzkopf verkehrt* (*kkv*) (Tonning et al., 2005), no Serp accumulation in the tracheal lumen was detected (Fig. 2G,G′), suggesting that chitin synthesis is essential for retention of Serp in the tracheal lumen.

### Haemolymph-derived Serp supports tracheal development

**Fig. 3.**
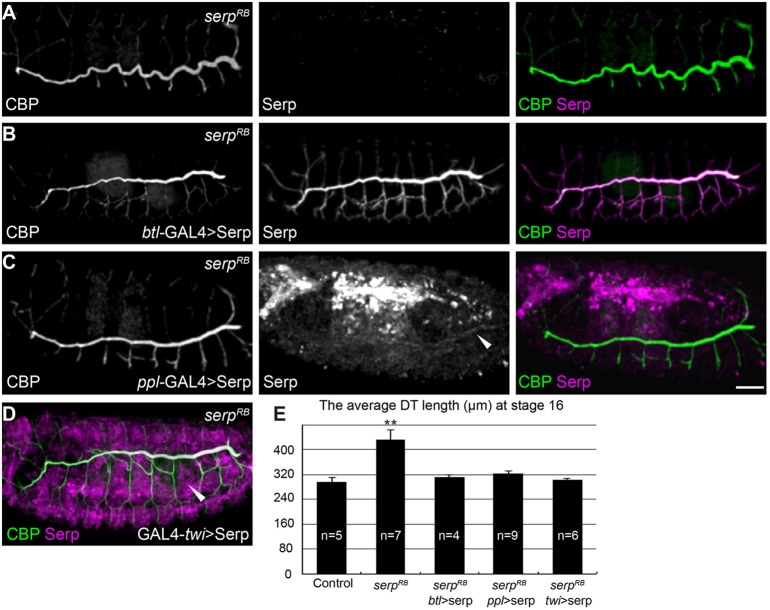
**Fat body-derived Serp rescues the tracheal tube length defect in *serp^RB^* mutants.** (A-C) Left, middle and right panels indicate CBP, anti-Serp and merged images, respectively. ** **(A) Stage 16 *serp^RB^* homozygous mutant embryos stained with anti-Serp and CBP to show the dorsal trunk (DT). (B-D) Stage-16 *serp^RB^* homozygous mutant embryos expressing full-length Serp by *btl*-GAL4 (B), *ppl*-GAL4 (C) and *twi-*GAL4 (D), respectively. White arrowheads in C show Serp accumulation in the tracheal lumen. White arrowhead in D indicates Serp production in the mesodermal tissue. (E) Quantification of DT length. Data are mean±s.e.m. **P<0.01 between *serp^RB^* and control by Student's *t*-test. Scale bar: 50 μm in A-D.

To determine whether Serp transport from the haemolymph to the tracheal lumen plays an active morphological role, we expressed full-length Serp in *serp* mutants. Expression of Serp by the trachea driver (*btl*-GAL4) rescued the over-elongated tube phenotype (Fig. 3A,B). Importantly, when Serp was expressed by *ppl-*GAL4 or *twi-*GAL4 in *serp* mutant embryos, similar levels of rescue effect were observed (Fig. 3C-E). These results indicated that the haemolymph-derived Serp is functional.

### Non-tracheal source of Serp contributes to normal tracheal development

**Fig. 4.**
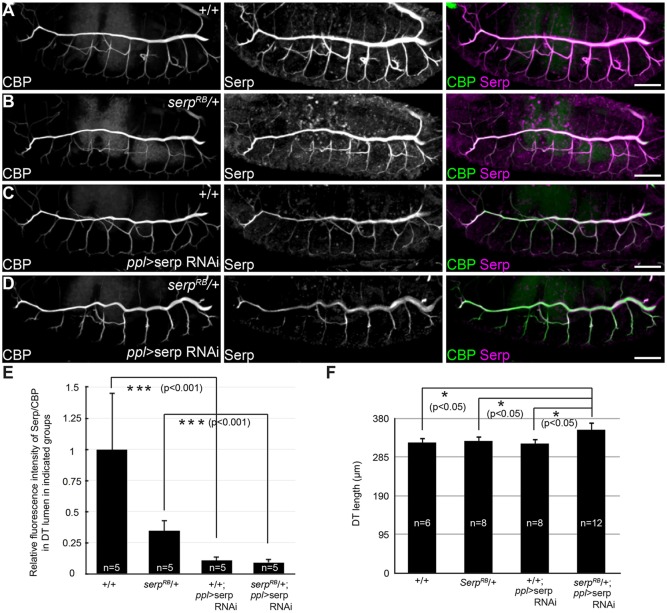
**Non-tracheal Serp is redundantly required for tracheal tube-length control.** (A-D) Left, middle and right panels indicate CBP, Serp and merged images, respectively. Stage 16 control (A), *serp^RB^* heterozygote (B), embryo expressing *serp* RNAi by *ppl*-GAL4 (C) and *serp^RB^* heterozygote expressing *serp* RNAi by *ppl*-GAL4 (D) were stained with CBP and anti-Serp. (E) Quantification of the relative fluorescence intensity of Serp/CBP in the tracheal lumen. Data are mean±s.e.m. (F) Quantification of DT length. Data are mean±s.e.m. *P<0.05, ***P<0.001 by Student's *t*-test. Scale bars: 50 μm.

Finally, we asked whether a non-tracheal source of Serp is required for normal tracheal development by UAS-Serp RNAi driven by *ppl-*GAL4. In a wild-type genetic background, *serp* knockdown with *ppl-*GAL4 reduced Serp levels in the tracheal lumen, but did not cause any defect in tube morphology (Fig. 4A,C,E,F; *ppl-*GAL4 is not expressed in the tracheal cells). Similarly, reducing the *serp* gene dose by half using a heterozygous *serp^RB^* had no effect on tracheal tube morphology (Fig. 4B,F). However, the combination of fat body-specific *serp* knockdown with reduced gene dose resulted in abnormally elongated tube (Fig. 4B,D,F), suggesting that Serp expressed in the fat body and trachea functions redundantly, and that fat body-derived Serp is only essential for tube morphogenesis when the trachea-derived product is limiting.

### The fat body is the source of an aECM protein

We here found that the apical ECM (aECM) component Serp is produced not only by tracheal cells lining the lumen, but also by mesodermal fat body cells. We propose that Serp synthesized in the fat body contributes to its accumulation in the tracheal lumen, based on the following evidence. First, *serp* transcripts were detected in fat body cells. Second, *serp* knockdown by *ppl-*GAL4 significantly decreased Serp protein levels in the tracheal lumen (Fig. 4E). Third, inhibition of the vesicle secretory pathway in embryos using BFA resulted in the intracellular accumulation of Serp in the fat body. Serp protein was also detected in the fat body of third-instar larvae (Stefan Luschnig, personal communication). We also found that expression of Serp by the mesodermal driver functionally complemented *serp* mutant tracheal phenotype, and low-level *serp* mRNA was detectable in internal tissues, including gut, raising the possibility that other internal organs also serve as sources of Serp.

### Translocation of Serp from the fat body to the tracheal lumen

Serp produced in the fat body is secreted into the haemolymph, taken up by tracheal cells, and then transcytosed to the lumen. We have previously shown that secreted luminal Serp is recycled in the tracheal cells and transported back to the lumen via retrograde trafficking (Dong et al., 2013). Once Serp is internalized from the basal side, it may be transported to the recycling pathway and then released into the lumen, where it is retained by binding to chitin (supplementary material Fig. S4). Thus, through retrograde recycling and transcytosis pathways, sufficient levels of Serp are maintained in the tracheal lumen, thereby facilitating the dynamic remodelling of the aECM that is essential for the regulation of tube morphogenesis.

### The fat body as a coordinator of systemic growth and patterning

The fat body supplies essential proteins required by other organs, such as yolk proteins for oogenesis (Telfer, 1961; Roth and Porter, 1964; Pan et al., 1969; Hagedorn and Fallon, 1973), collagen IV for imaginal discs (Pastor-Pareja and Xu, 2011) and xanthine dehydrogenase for eye pigmentation (Reaume et al., 1989). Proteins synthesized and/or stored in the fat body of ants were found in the cuticle and eggshell (Roma et al., 2006, 2008). Our findings suggest that Serp should be added to the increasing repertoire of proteins that the fat body supplies to other organs. The transport of Serp from the fat body is unique in that the protein reaches the tracheal lumen and regulates tracheal tube length. Thus, our results, taken with earlier findings, support the hypothesis that the fat body plays essential roles in the development of multiple organs through the production and secretion of numerous proteins.

## MATERIALS AND METHODS

### Fly strains and genetics

*shrub^4^*/CyO and UAS-Shrub-GFP were gifts from Fenbiao Gao (Sweeney et al., 2006); UAS-Serp-CBD-GFP, UAS-Serp-GFP and *serp^RB^* were gifts from Stefan Luschnig (Luschnig et al., 2006); UAS-ANF-GFP was a gift from Christos Samakovlis (Tsarouhas et al., 2007); *rab9^56^* and *rab9^199^* have been described previously (Dong et al., 2013); *btl*-Moesin-RFP was a gift from Markus Affolter (Ribeiro et al., 2004); UAS-*shi* K44A, *svp^1^*, *twi-*GAL4 and *kkv^1^* was from the DGRC in Kyoto; *ppl*-GAL4 (Colombani et al., 2003) and *cg*-GAL4 (FBti0027802) were gifts from Takashi Nishimura (RIKEN CDB, Kobe, Japan). The *serp* RNAi (v15466) strain was from the Vienna RNAi Center.

### Immunofluorescence and antibodies

The primary antibodies were: mouse monoclonal anti-Svp [1:10, a gift from Yasushi Hiromi (Kanai et al., 2005)]; guinea pig anti-Uif [1:500, a gift from Robert E. Ward (Zhang and Ward, 2009)]; and rabbit anti-Serp (1:300, a gift from Stefan Luschnig). The chitin-binding probe (CBP) (1:50) was prepared from a bacterial expression construct according to the protocol provided by Yinhua Zhang (New England Biolabs).

### *In situ* hybridization

The *serp* probe was designed as described previously (Luschnig et al., 2006). The sequence was cloned into pBluescript II KS and then digested with *Xho*I or *Eco*RI to create the template for *in vitro* transcription using T7 and T3, respectively, to produce sense and antisense probes. Whole-mount wild-type embryos were fixed using 4% paraformaldehyde in PBS. *In situ* hybridization was conducted using DIG-labelled sense and antisense probes, following a standard protocol. After hybridization, stage 16 embryos were cut transversely with needles, and then *serp* mRNA expression was analysed.

### Serp expression by laser heat shock

Serp-CBD-GFP was digested by *Eco*RI and *Xba*I from UAS-Serp-CBD-GFP construct (Luschnig et al., 2006), and then was inserted into *Eco*RI and *Xba*I-digested pCaSpeR-hs vector. HS-Serp-CBD-GFP plasmid was injected into *y^1^ w^63C1^*; delta2-3 embryos to obtain a transgenic line. The trachea-surrounding tissues of embryos carrying HS>eGFP, HS>Serp-CBD-GFP and *btl*>Moesin-RFP (Fig. 1E) or HS>Serp-CBD-GFP alone (supplementary material Movie 1) were illuminated by infrared laser for transient induction of Hsp70 promoter (Kamei et al., 2009; Miao and Hayashi, 2014).

### Drug treatment

The embryos were dechorionated and incubated with heptane for 30 s, and then washed in a PBS buffer containing 0.5 mM CaCl_2_ and 0.5 mM MgCl_2_ for 30 s. The embryos were then incubated in 20 μg/ml brefeldin A in PBS buffer for 180 min. Brefeldin A was from Cell Signaling (catalogue number 9972) and was dissolved in DMSO.

### Confocal images and data analysis

Images were acquired using a laser-scanning confocal microscope (FV1000, Olympus) equipped with 60× oil- and water-immersion objectives. Malma software (Kagayaki Kato, unpublished) was used to adjust the images and to measure the fluorescent intensity. The dorsal trunks (DTs) were measured in fixed embryos (5 to 12) using CBP staining and analysed with Fiji-Image J. The expression level of Serp in different genotypes was quantified as anti-Serp fluorescence intensity normalized by the level of CBP staining. Student's two-tailed unpaired *t-*test with equal variance was used to assess statistical significance.

## Supplementary Material

Supplementary Material
