# Supplementary material for: A fat body-derived apical extracellular matrix enzyme is transported to the tracheal lumen and is required for tube morphogenesis in Drosophila
Source: Development. 2014 Nov;141(21):4104–9. doi: 10.1242/dev.109975 (PMC4302886; doi:10.1242/dev.109975)
Supplement: Supplementary Material [file supp_141_21_4104__index.html]

Supplementary Material 

# A fat body-derived apical extracellular matrix enzyme is transported to the tracheal lumen and is required for tube morphogenesis in *Drosophila*

## DEV109975 Supplementary Material

**Files in this Data Supplement:**

- Supplementary Material
