# Supplementary material for: A fat body-derived apical extracellular matrix enzyme is transported to the tracheal lumen and is required for tube morphogenesis in Drosophila
Source: Development. 2014 Nov;141(21):4104–9. doi: 10.1242/dev.109975 (PMC4302886; doi:10.1242/dev.109975)
Supplement: Supplementary Material [file supp_141.21.4104_DEV109975supp.pdf]

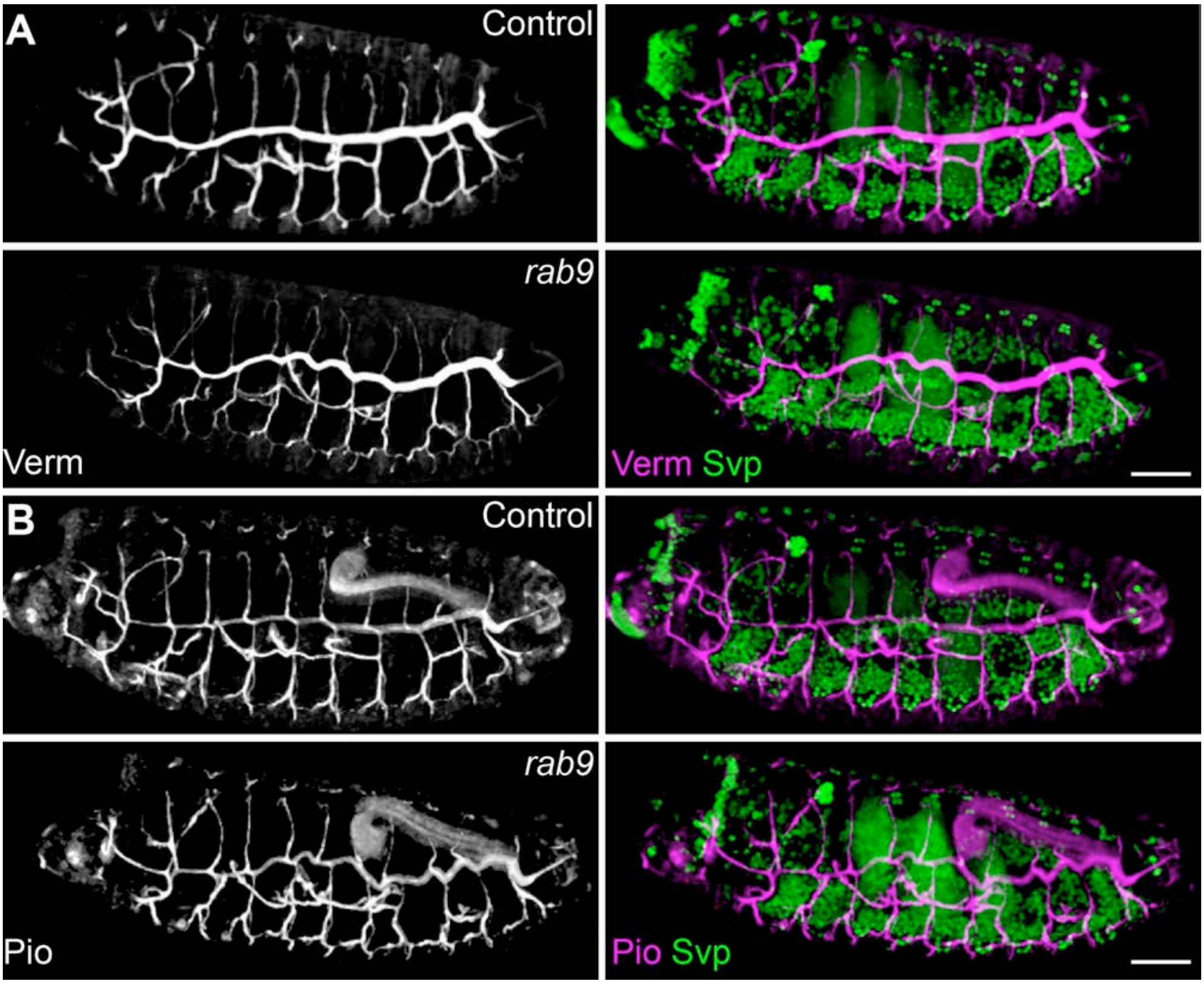

Fig. S1. Localization of luminal proteins in *rab9* mutants.

(A-B) Stage-16 control and *rab9* homozygous mutant embryos were stained with anti-Verm (A) and anti-Pio (B) respectively. All the samples were stained with anti-Seven-up (Svp) to indicate the fat body cells in the embryos. Both Verm and Pio were detected in the tracheal lumen but not in the fat body. Scale bars: 50 μm.

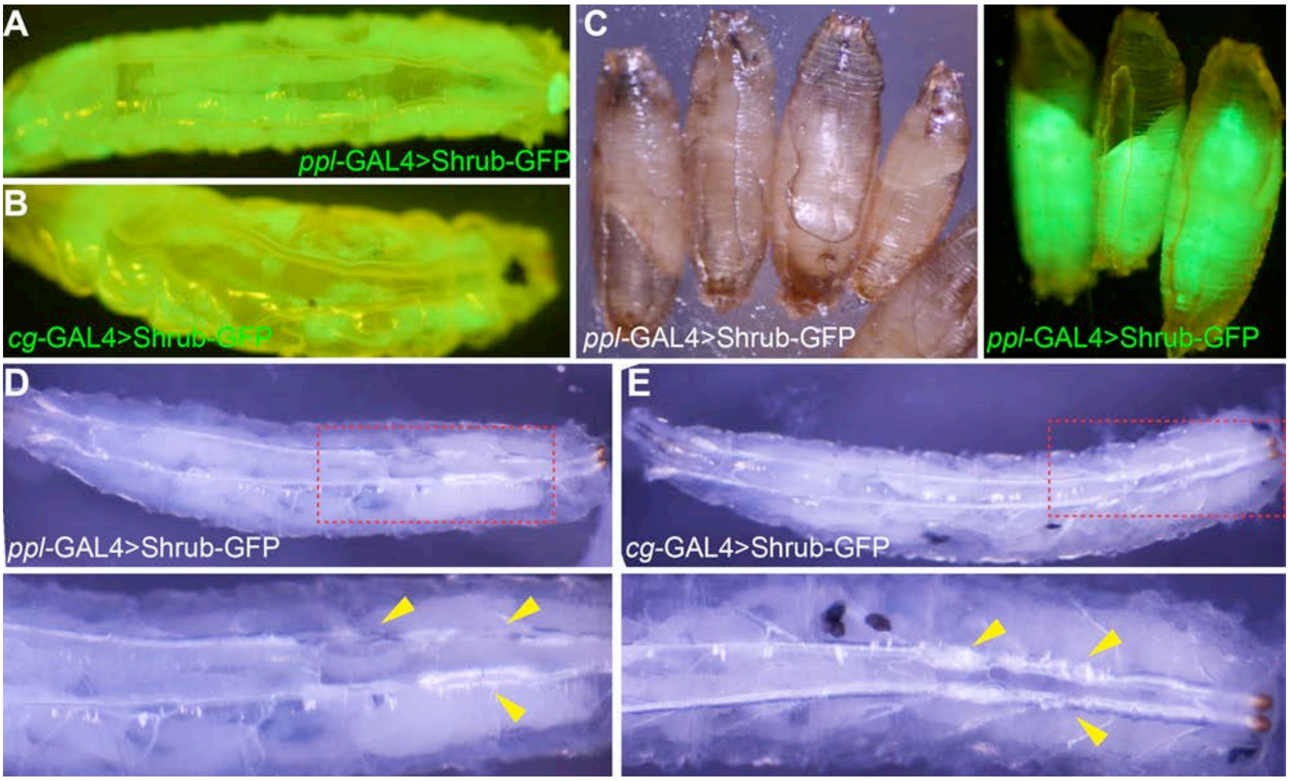

Fig. S2. Interference with ESCRT III function in the fat body causes morphological defects in the larval tracheal system.

(A-B) Third-instar larvae expressing dominant-negative Shrub-GFP using the fat body drivers *ppl*-GAL4 (A) and *cg*-GAL4 (B). (C) Dead pupae of *ppl*-GAL4>Shrub-GFP. (D-E) Partially collapsed tracheal tubes in third-instar larvae expressing dominant-negative Shrub-GFP with the *ppl*-GAL4 (D) and *cg*-GAL4 (E) drivers. Yellow arrowheads in the bottom panels (magnified views) indicate the collapsed tracheal tube.

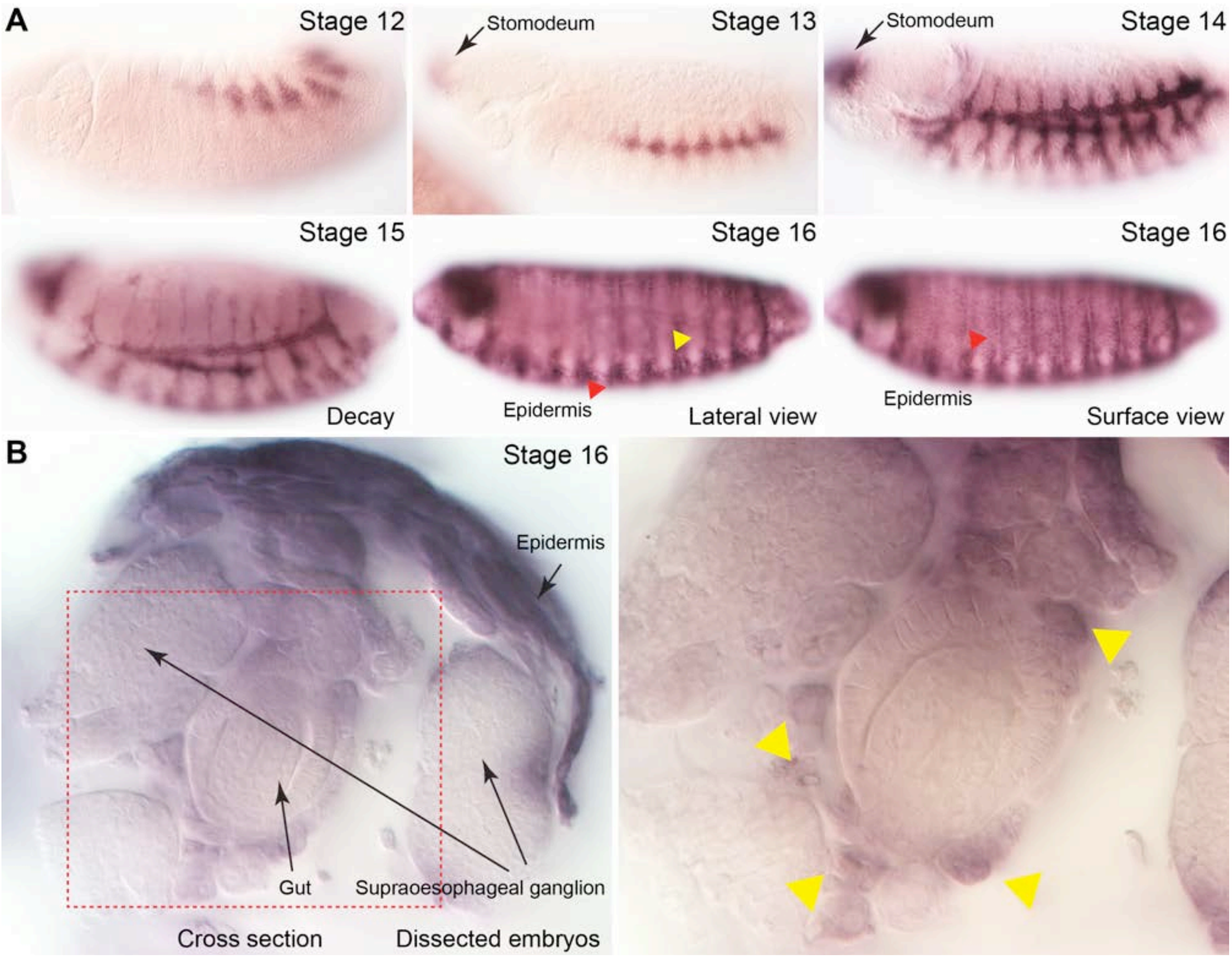

Fig. S3. Expression of *serp* mRNA.

(A) In situ hybridization of *serp* mRNA. Transcript of *serp* was detected in the tracheal cells at stage 12 and had increased by stage 14. *serp* expression in the trachea was reduced at stage 15 and was very weak by stage 16 (yellow arrowhead). *serp* mRNA was detected in the stomodeum at stage 13 and later stages (black arrows). At stage 16, *serp* expression in tracheal cells was reduced (lateral view) and strongly detected in the epidermis (red arrowheads). (B). *serp* mRNA was detected in the fat body cells. Cross-section of the anterior part of a stage-16 embryo at the level of the oesophagus and supraoesopharyngeal ganglion. Dorsal epidermis is located at upper right. The yellow arrowheads in the inset indicate *serp* mRNA expression in the fat body cells.

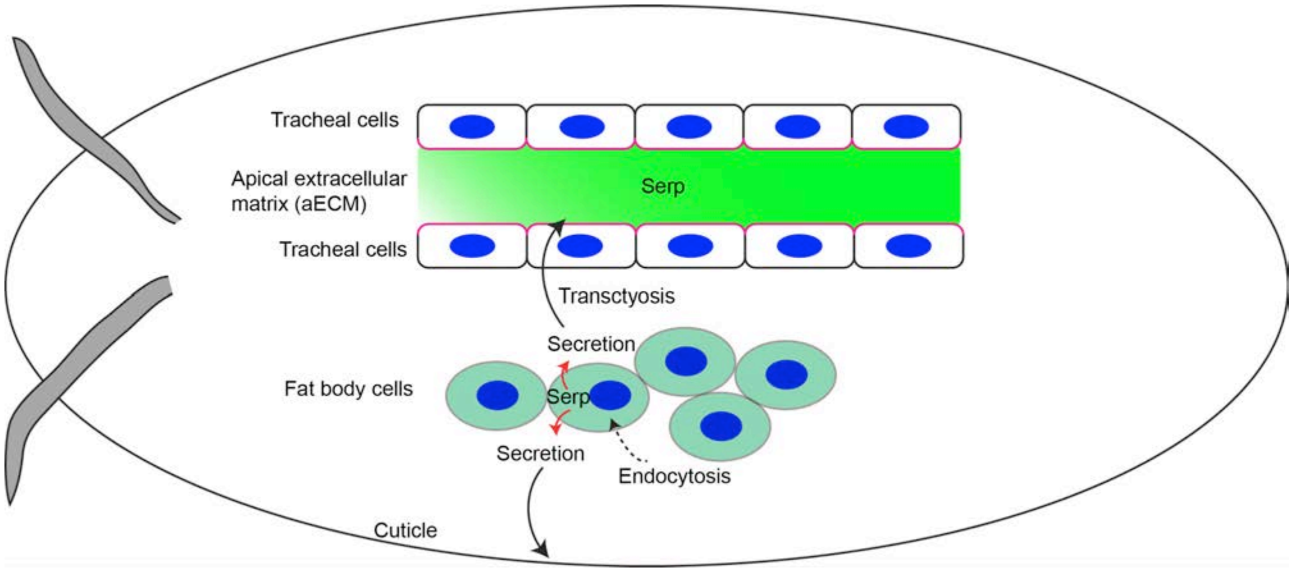

Fig. S4. Model for Serp translocation from the fat body to the tracheal lumen.

The fat body is a Serp-producing and re-cycling organ. Synthesized or recycled Serp is secreted into the haemolymph, and then transcytosed across the tracheal cells to the tracheal lumen for aECM modification.

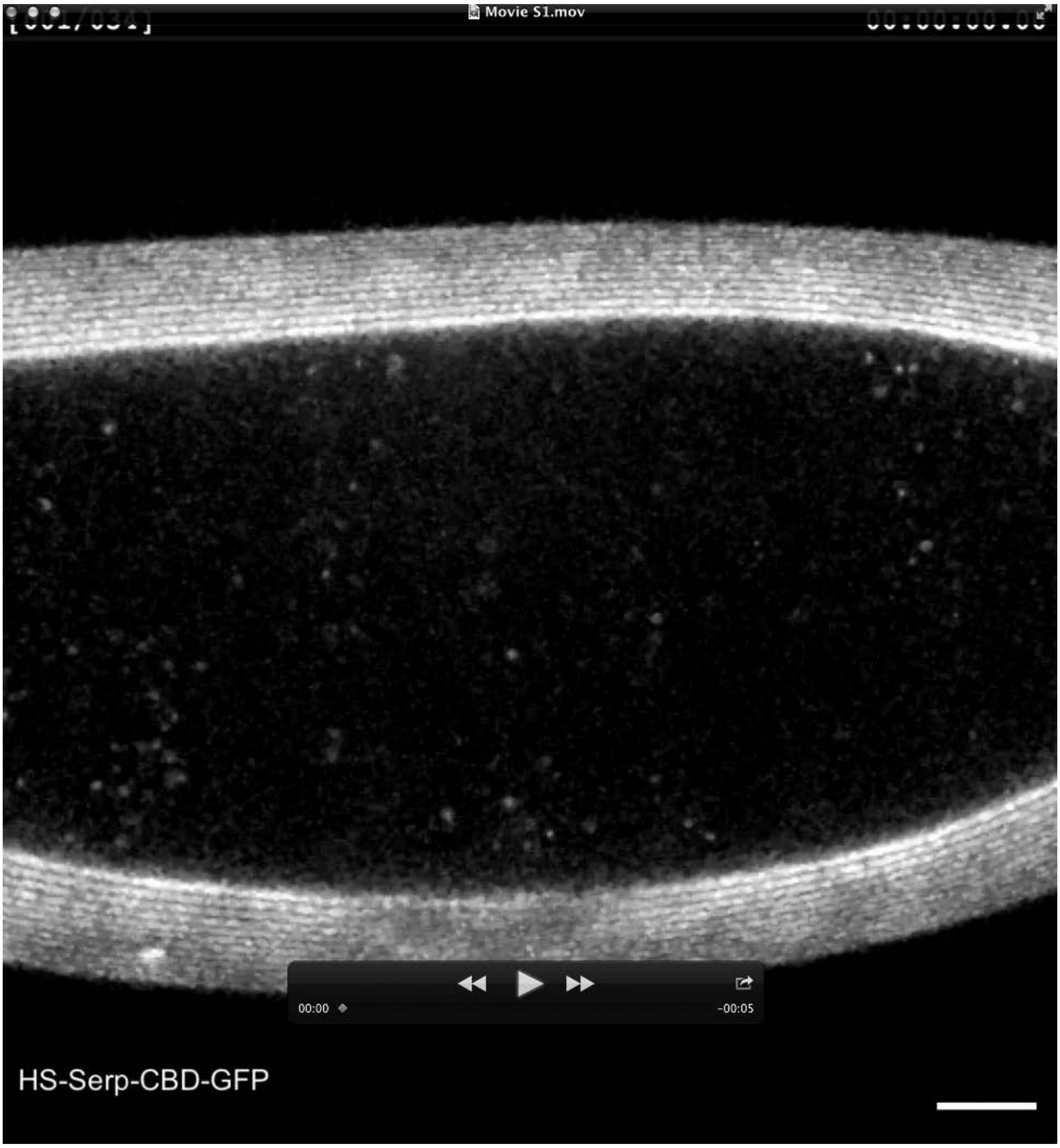

Legend for Supplemental Movie: Movie S1

Induction of Serp-CBD-GFP by localized IR laser heat shock. An embryo carrying HS>Serp-CBD-GFP was heat-shocked at non-tracheal tissues by IR laser at later of stage 14. After 90 minutes, GFP signal presents in the lumen of dorsal trunk. A cluster of bright spots appearing at 24 minutes is yolk auto fluorescence. Bar represents 20 μm.
